# Utility of 1.5 Tesla MRI Scanner in the Management of Small Sample Sizes Driven from 3D Breast Cell Culture

**DOI:** 10.3390/ijms25053009

**Published:** 2024-03-05

**Authors:** Wiesław Guz, Rafał Podgórski, David Aebisher, Adrian Truszkiewicz, Agnieszka Machorowska-Pieniążek, Grzegorz Cieślar, Aleksandra Kawczyk-Krupka, Dorota Bartusik-Aebisher

**Affiliations:** 1 Department of Diagnostic Imaging and Nuclear Medicine, Medical College of the University of Rzeszow, 35-310 Rzeszów, Poland; wguz@ur.edu.pl; 2 Department of Biochemistry and General Chemistry, Medical College of the University of Rzeszow, 35-310 Rzeszów, Poland; rpodgorski@ur.edu.pl; 3 Department of Photomedicine and Physical Chemistry, Medical College of the University of Rzeszow, 35-310 Rzeszów, Poland; daebisher@ur.edu.pl (D.A.);; 4 Department of Orthodontics, Faculty of Medical Sciences in Zabrze, Medical University of Silesia, 40-055 Katowice, Poland; apieniazek@sum.edu.pl; 5 Department of Internal Diseases, Angiology and Physical Medicine, Centre for Laser Diagnostics and Therapy, Medical University of Silesia in Katowice, Batorego 15, 41-902 Bytom, Poland; cieslar1@tlen.pl

**Keywords:** magnetic resonance imaging, coil, breast cancer cells, 1.5 Tesla, in vitro MRI, bioreactor, 3D, glycosaminoglycan, fixed-charge density

## Abstract

The aim of this work was to use and optimize a 1.5 Tesla magnetic resonance imaging (MRI) system for three-dimensional (3D) images of small samples obtained from breast cell cultures in vitro. The basis of this study was to design MRI equipment to enable imaging of MCF-7 breast cancer cell cultures (about 1 million cells) in 1.5 and 2 mL glass tubes and/or bioreactors with an external diameter of less than 20 mm. Additionally, the development of software to calculate longitudinal and transverse relaxation times is described. Imaging tests were performed using a clinical MRI scanner OPTIMA 360 manufactured by GEMS. Due to the size of the tested objects, it was necessary to design additional receiving circuits allowing for the study of MCF-7 cell cultures placed in glass bioreactors. The examined sample’s volume did not exceed 2.0 mL nor did the number of cells exceed 1 million. This work also included a modification of the sequence to allow for the analysis of T_1_ and T_2_ relaxation times. The analysis was performed using the MATLAB package (produced by MathWorks). The created application is based on medical MR images saved in the DICOM3.0 standard which ensures that the data analyzed are reliable and unchangeable in an unintentional manner that could affect the measurement results. The possibility of using 1.5 T MRI systems for cell culture research providing quantitative information from in vitro studies was realized. The scanning resolution for FOV = 5 cm and the matrix was achieved at a level of resolution of less than 0.1 mm/pixel. Receiving elements were built allowing for the acquisition of data for MRI image reconstruction confirmed by images of a phantom with a known structure and geometry. Magnetic resonance sequences were modified for the saturation recovery (SR) method, the purpose of which was to determine relaxation times. An application in MATLAB was developed that allows for the analysis of T_1_ and T_2_ relaxation times. The relaxation times of cell cultures were determined over a 6-week period. In the first week, the T_1_ time value was 1100 ± 40 ms, which decreased to 673 ± 59 ms by the sixth week. For T_2_, the results were 171 ± 10 ms and 128 ± 12 ms, respectively.

## 1. Introduction

Over the last decade, research on cellular metabolism has elucidated the various valuable biological actions of glycosaminoglycans (GAGs) in cell development, cellular homeostasis and pathological processes [1]. Glycosaminoglycans are negatively charged linear polysaccharides frequently found in the extracellular matrix (ECM) conjugated with proteins and/or forming proteoglycans (PGs). Glycosaminoglycans play important structural and regulatory roles in the ECM. Glycosaminoglycans are involved in cellular signaling processes governing tissue growth and development. Important types of GAGs that differ in chemical composition, structure and function include the non-sulfated hyaluronan, the sulfated heparan sulfate and the closely related heparin, the sulfated chondroitin sulfate and the related dermatan sulfate, and keratan sulfate [2]. 

In this research, we captured aspects of the physiological adaptation of living cells in three-dimensional (3D) breast cell cultures by MRI. A network of proteins and polysaccharides (e.g., collagens, elastin, fibronectin, laminins, glycoproteins, proteoglycans and GAGs) are generated intracellularly by cells and secreted by exocytosis [3]. Naturally, cells contain primary protein and GAG associated via covalent and noncovalent molecular interactions and these compounds form long carbohydrates in breast cancer tissue [4]. Due to their negative charge, they bind with various ions such as the contrast agent Gd-DTPA^2−^ [5]. 

In this study, we present magnetic resonance imaging (MRI) of cells in a 3D breast cancer cell culture using a 1.5 Tesla (T) apparatus to image the biochemistry and biological phenomena of cells, e.g., GAG content. We expected that the concentration of GAG would be increased during a 6-week growth period of an in vitro 3D cell culture. Glycosaminoglycan concentration was possible to determine using the fixed-charge density (FCD) values [6] obtained by MRI measurements with Gd-DTPA^2−^.

Magnetic resonance imaging is a key imaging methodology that is increasingly being used for preclinical in vitro and clinical in vivo studies of cellular processes [7,8]. Magnetic resonance imaging produces images of pathological changes in humans, animals and plants in two-dimensional (2D) and three-dimensional geometry. Also, by the same MR systems, we can obtain spectra by using magnetic resonance spectroscopy (MRS) pulse sequences. This opportunity allows for the analysis of many types of biological samples, e.g., cancer cell cultures. In vivo MRI allows for the assessment of the structure of internal organs, metabolic pathways and disorders in organs, tissues and cells. In vitro and in vivo MRI allows for the measurement of images, spin–lattice (T_1_) and spin–spin (T_2_) relaxation times, and spectra during one measurement with one sample set up in the machine. Thus, MRI methodology allows for efficient three-dimensional imaging and sample characterization. The use of 3D cancer cell cultures for metabolomic studies enables in vivo investigations on a living organism. 

The aim of this study was to use T_1_ spin–lattice and T_2_ spin–spin relaxation time to develop a methodology for the imaging of 3D cancer cell cultures in vitro. In addition to this, the GAG concentration was estimated based on T_1_ spin–lattice relaxation time in cells and media.

It is known that two-dimensional (2D) surfaces do not mimic the differential structures observed in vivo [9]. Magnetic resonance imaging offers non-invasive in vivo and in vitro tissue and cell imaging with high sensitivity and time resolution [10]; however, an in vitro MRI of cells requires a sample density similar to that used under in vivo conditions. 

The MRI signal depends on the biochemical properties of the tested cells and the physical parameters selected for testing. In MCF-7 breast cancer cells, Her-2 overexpression is associated with a higher growth rate of neoplastic cells, which is directly related to a higher culture density. These Her-2 receptors provide an opportunity for active drug targeting [11]. Cell culture studies enable imaging of dynamic and kinetic changes by studying the T_1_ and T_2_ relaxation times [12,13,14]. An efficient 3D cell culture model of preclinical testing must reproduce the human tumors and mimic in vivo conditions [15,16,17,18]. 

We used a 1.5 T MRI scanner to image cell cultures and develop an application that can measure both T_1_ and T_2_ relaxation times. This method was developed in the MATLAB package and is able to provide statistics, function approximations, data interpolation and use of the architecture of DICOM files. We verify calculations by using an algorithm based on the results from phantom measurements. The presented model of 3D breast cancer cells attempts to mimic in vivo conditions. Our hypothesis is that MRI relaxation time can monitor breast cancer cell growth by measuring the influence of Her-2 expression and intracellular matrix development on proton mobility in MCF-7 Her-2 positive cells. 

## 2. Results

### 2.1. Three-Dimensional MCF-7 Cells

**Figure 1:**
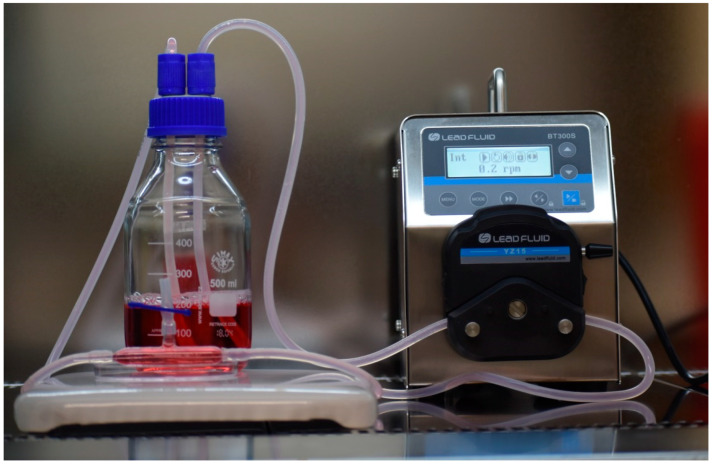
Bioreactor setup.

Studies on 3D cell cultures allow the use of MRI to produce cellular images and measurements of relaxation times. In addition, a new coil with appropriate parameters was designed to be used in our 1.5 T MR system. The development of 3D cell cultures allows for the preparation of new protocols which may lead to new methodologies of treatment detection at 1.5 T. Three-dimensional breast cancer cells remained in a bioreactor throughout the MR measurements without changes in setup. The circulation rate of the media was chosen to keep the cells in the space of the bioreactor (Figure 1). In the bioreactor and the loop, the circulation rate of media was reduced during both inoculation and growth of the cell culture. This flow reduction was achieved by directing the ICcirc pump in the opposite direction to the ICinlet pump allowing cells to remain in the bioreactor. Immunohistochemistry (IHC) is a special staining process performed on fresh or frozen breast cancer tissue removed during biopsy. Immunohistochemistry was used to determine if the cancer cells had expressed Her-2 receptors on their surface. This information plays a critical role in treatment planning. With too many Her-2 receptors, the cells receive too many signals telling them to grow and divide. The IHC test gives a score of 3+ that measures the amount of Her-2 receptor protein on the surface of cells in a breast cancer tissue sample. This score was confirmed by flow cytometry. With the use of a bioreactor, we grew 3D breast cancer cells at a density of 10^9^–10^10^ cells/mL. 

### 2.2. Cellular MRI

In the first week of growth, the cell formed a thin layer of tissue on the lower surface of the bioreactor and around the fibers. At this stage, the cells were easily disrupted during extraction from the bioreactor. After six weeks of growth, the cells almost filled extra capillary space. The pH range was maintained in the extra-capillary space through the duration of experiments from 6.8 to 7.0. Table 1 below presents the averaged weekly change of cell numbers in six bioreactors. 

**Figure 2:**
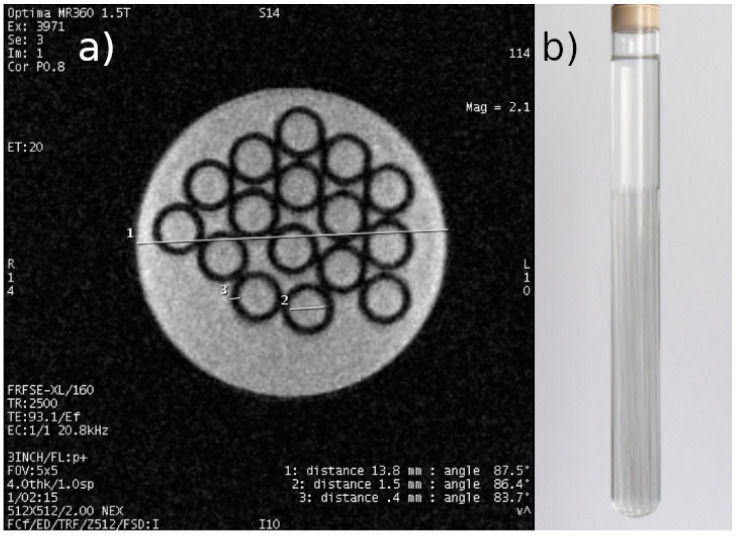
(**a**) Representative result of scanning a phantom of a test tube filled with demineralized water with 16 capillaries immersed in it, with an actual external diameter of 2.3 mm and a wall thickness of 0.4 mm. Inner diameter of the test tube: 13.8 mm (actual set values determined with a caliper with an accuracy of 0.05). Scanning parameters were as follows: FOV: 5 cm, acquisition matrix: 512 × 512, TE: 93.1 ms, TR: 2500 ms, layer thickness: 4 mm. (**b**) Photo of the phantom.

^1^H MRI of breast cancer cells in vitro was developed by using the following category of our research requirements at 1.5 T:

(1) Development of a numerical application measuring relaxation times and its implementation in the MATLAB package (statistics, function approximation, data interpolation, use of DICOM files).

(2) Preparation and performance of ^1^H MRI measurements with standard substances.

(3) Verification of the calculation algorithm based on the literature and the results of phantom measurements.

(4) Preparation of tumor MCF-7 cell cultures the density of which will enable qualitative and quantitative ^1^H MRI measurements in order to develop appropriate databases. Figure 2 shows the phantom MRI scan. 

Over time, both T_1_ and T_2_ relaxation times decreased significantly for MCF-7, from 1100 ± 40 ms to 795 ± 31 ms and from 171 ± 10 ms to 137 ± 21 ms, respectively. Values of T_1_, T_2_, and time after inoculation are presented in Table 2 for MCF-7 cells.

**Figure 3:**
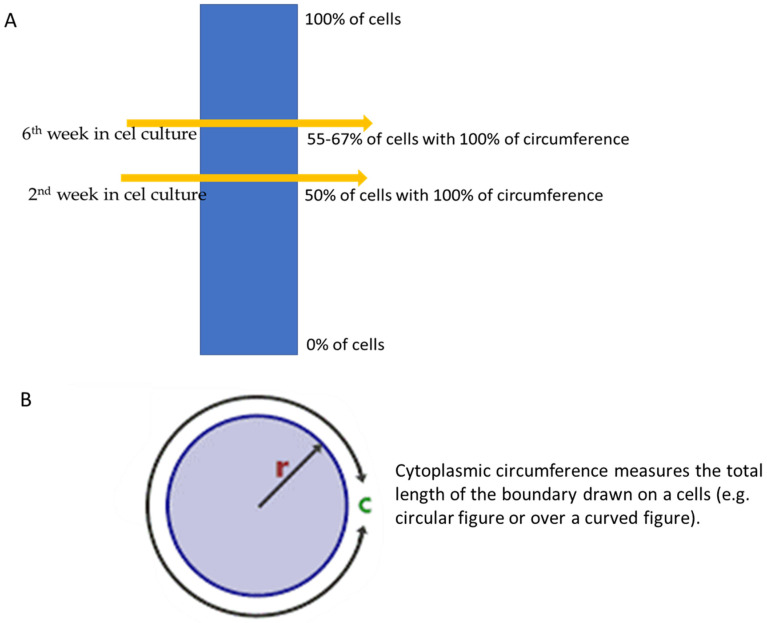
(**A**,**B**) Changes in cytoplasmic circumference during 6 weeks, where r is the circle radius and c is the circumference of a circle.

T_1_ relaxation time of MCF 7 cells decreased over 6 weeks in culture by approximately 40% and the value of T_2_ by 20%. Her-2 expression was confirmed based on the IHC test after two and six weeks in culture. In the second week in culture, the detection of Her-2 showed that definitive staining of the membrane over 100% of the cytoplasmic circumference was noticed in 50% of the neoplastic cell population, and after the sixth week, the cytoplasmic circumference was noticed in 55–67% of MCF-7 cell cultures (Figure 3).

**Figure 4:**
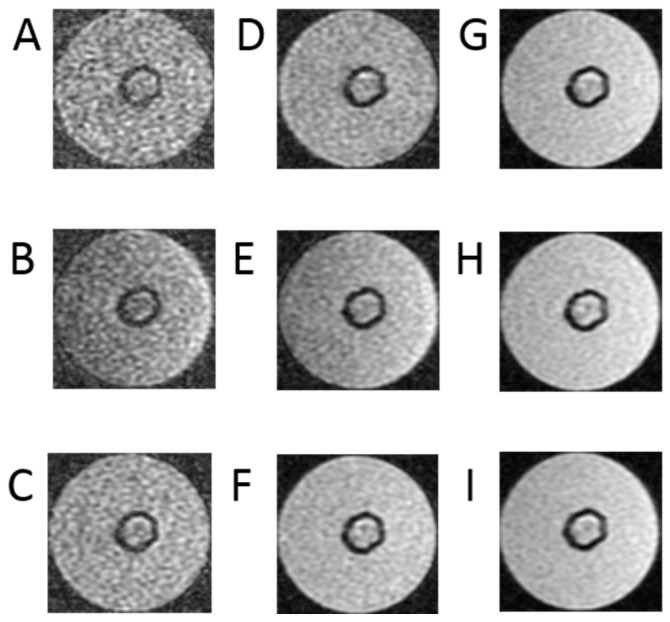
Images of T1 relaxation time measurements as follows: (**A**) images with TR = 500 ms, (**B**) images with TR = 700 ms, (**C**) images with TR = 1000 ms, (**D**) images with TR = 1500 ms, (**E**) images with TR = 2000 ms, (**F**) images with TR = 3000 ms, (**G**) images with TR = 5000 ms, (**H**) T images with R = 10,000 ms, (**I**) images with TR = 15,000 ms, TE = 3 ms for bioreactor.

Figure 4 shows the border of the area where the cells are located in the bioreactor. It can be stated that the fluid constituting the culture medium has a much longer T_1_ time compared to the cell area. The calculated time T_1_ takes values up to 1100 ± 40 ms. The solid black background around the grey images of cells are the places where the R2 coefficient does not exceed 0.4, which indicates a poor fit. To remove noise from the image, the T_1_ values determined by R2 below 0.4 have been replaced with 0.

Figure 4 presents cross-sectional scans of the tested bioreactor. The presented images are a series of data that are the basis for determining the longitudinal relaxation time—T_1_ of the examined space. They were performed using clinically used, and in this case modified, sequences. The signal acquisition took place with an echo time of TE = 3 ms. Such a short echo time allows us to minimize the influence of the exponential component containing the quotient of TE and T_2_ time, which reduces the useful signal as TE increases. The study was performed for a series of TR repetition times. The test was performed for a series of TR repetition times. They were, respectively, 500 ms, 700 ms, 1000 ms, 1500 ms, 2000 ms, 3000 ms, 5000 ms, 10,000 ms and 15,000 ms. The use of a very long time of 15,000 ms requires some explanation—this is due to the fact that in the SR method, the T_1_ time is defined as a drop to 63% of the maximum value. Reducing the time may increase the error in determining the T_1_ parameter. Generally, it is recommended that the TR time be equal to or greater than five times the T_1_ time. A large number of measurement points increases the approximation accuracy. It should be added that in this case, there is no absolute need to minimize the examination time, as is the case with MRI examinations in patients. In this group of images, the scan obtained with the longest time is characterized by the largest signal in relation to the noise. The visibility of noise increases as the TR time decreases.

**Figure 5:**
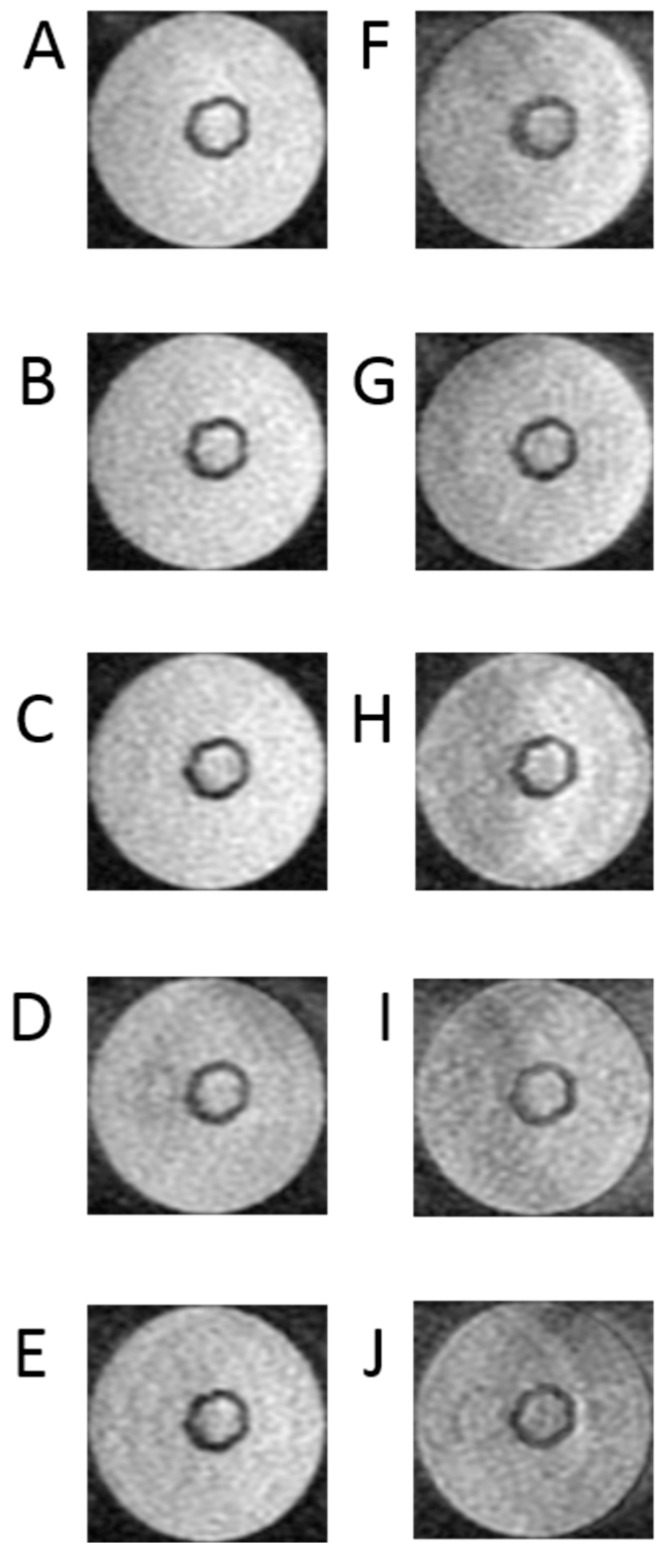
Example of one set of T2 relaxation time of 3D breast cancer cells in hollow fiber bioreactor with pulse sequence parameters as follows: (**A**) TE = 10 ms, (**B**) TE = 20 ms, (**C**) TE = 26.2 ms, (**D**) TE = 42 ms, (**E**) TE = 68 ms, (**F**) TE = 85 ms, (**G**) TE = 102 ms, (**H**) TE = 130 ms, (**I**) TE = 160 ms, (**J**) TE = 200 ms, TR = 10,000 ms. The same parameters were used for each bioreactor (*n* = 6).

Figure 5 shows nine sscans selected in the acquired sequences to determine the T_2_ time. 

Figure 5 presents a series of images necessary to determine the transverse relaxation times of the examined object cross-section. In this case, image acquisition was performed at TR = 1000 ms. This time was selected experimentally to minimize the influence of the exponential term containing the product of the TR times and the T1 time. Also, in this case, to minimize errors related to approximation, a larger number of images was recorded, namely −10. The acquisition included images at echo times TE = 10 ms, 20 ms, 26.2 ms, 42 ms, 68 ms, 85 ms, 102 ms, 130 ms, 160 ms, and 200 ms. Analyzing the obtained images, it is clearly visible that those obtained with short TE are characterized by a much better signal-to-noise ratio, which is fully understandable. These images, obtained with short echo times, are the best quality images.

**Figure 6:**
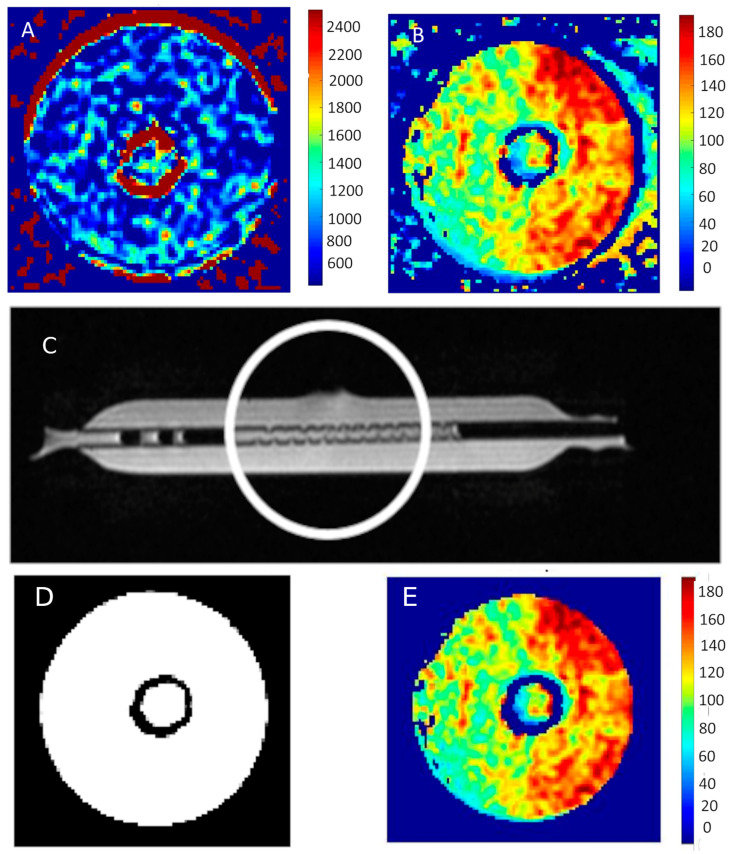
(**A**) T_1_ map and (**B**) T_2_ map of MCF-7 cells. (**C**) Bioreactor prepared for cell culture, the circle indicates the place where the culture is planned. (**D**) Response of the neural network classifying pixels into two groups. (**E**) The result of the procedure of the neural network is the removal of interferences from the image presented T2 map of time distribution.

Figure 6 shows an example of the map distribution of T_1_ and T_2_ times in the tested bioreactors containing MCF-7 breast cancer cells.

Figure 6D,E show images exposed to the neural network. As a result of its operation, both artifacts and noise itself are removed. This can be seen by comparing the image showing the distribution of T_2_ relaxation times in Figure 6B with the same cross-section shown in Figure 5C. 

Figure 6D presents the response of the neural network classifying individual pixels into two groups—the first one containing noise and artifacts and the second one showing the examined area—the bioreactor area. The neural network itself was designed to analyze a signal that changes over time. Such a network can differentiate the time course. The proposed neural network as well as its validation have been reported [19,20]. In the work above, it was also used by researchers to eliminate noise and reduce the number of calculations by eliminating noisy pixels.

The images clearly show the area with shorter T_1_ and T_2_ times in the area occupied by the cells. The measurement was taken in the center of the area representing the cells. It should be noted that the image contains artifacts—they appear in the area of the walls of the test tubes, as well as at the liquid–air interface.

The results of the MRI after adding Gadovist contrast (1 mg/1 mL) are presented below. Proton MR image of MCF-7 breast cancer cells surrounded the fiber (7 weeks after inoculation).

This MRI research tested the hypothesis that T_1_ and T_2_ relaxation times results correlate with the biochemical composition of breast cancer cells in 3D geometry. Just outside the fiber walls, we find high proton density with relatively low mobility. Mobility increases with distance from the hollow fiber within the growth, corresponding to differences in cell size and density. The volume and cellularity increased during the bioreactor development over six weeks; this was accompanied by changes in MR properties, including relaxation times and water diffusion. These measurements just confirm a pre-established Her-2 expression in the MCF-7 cell line.

Our results indicate that both Her-2 impact and the main constituents of the cellular matrix in a 3D culture system have an influence on T_1_ and T_2_ relaxation times by decreasing their values.

**Figure 7:**
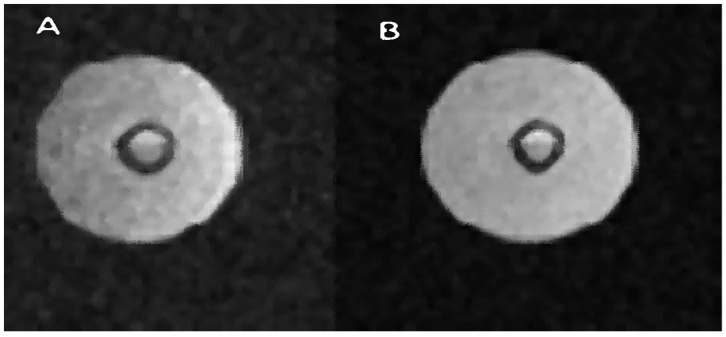
Example of bioreactor measurements with contrast agent Gadovist. Bright areas represent regions with a relatively high concentration of water. A spin–echo pulse sequence was used, with the following parameters: for (**A**) TR/TE 8000/16.5 ms; FOV, 3 × 3 cm and for (**B**) TR/TE 10,000 20 ms.

**Figure 8:**
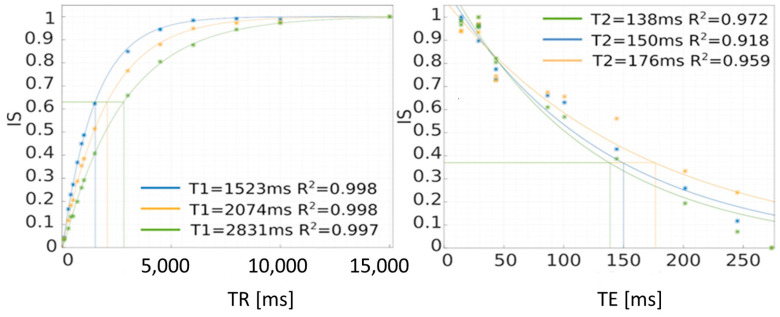
Method of determining T1 and T2 relaxation times using the saturation recovery method (SR). IS—intensity signal.

Figure 7 shows two images. Image A is a cross-sectional presentation of the bioreactor when it does not contain Gadovist. Image B, on the other hand, is a presentation in which this contrast agent was used. The difference you can notice is that the B image is enhanced. With a very similar brightness of the part containing a liquid with a large amount of water, the image of the air space and therefore the background noise were suppressed. This proves that the difference in signals between the water and air parts in images A and B is much greater for image B. The contrast agent shortens the T_1_ time and, therefore, performing two scans at constant TR will brighten the image in which the T_1_ time will be shorter. This relationship is shown in Figure 8, where for constant TR, the curves for shorter T_1_ times are steeper compared to the curves for longer T_1_ times. Figure 7 contains a presentation of the bioreactor for FOV = 5 cm, while the description states that the area of interest is 3 cm—a window of approximately 3 cm was used to present the bioreactor.

The FCD measurements showed increased concentrations of GAG (Table 3).

**Figure 9:**
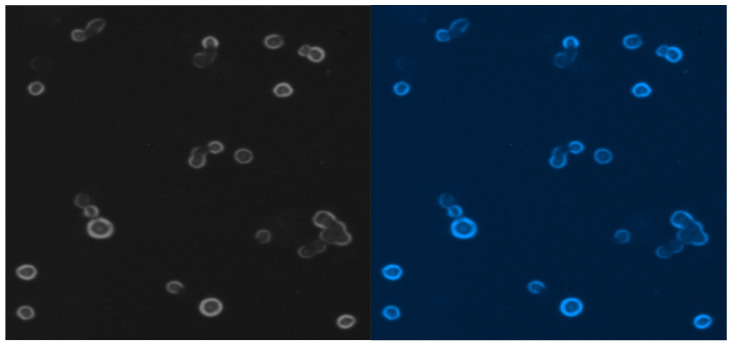
Free MCF-7 cells from liquid media cultured by 7 weeks.

Figure 9 presents MCF-7 cells labeled with blue fluorescent conjugate and only viable cells are visible after blue probe uptake.

## 3. Discussion

GAGs are critical for developmental processes and the maintenance of healthy tissues and biochemical processes [21]. The study by Emerman describes the types and distribution of GAGs accumulated by normal and malignant human mammary epithelial cells in primary culture during their exponential and stationary phases of growth [22]. GAGs play an important role in tissue organization through interactions with a diverse range of proteins, growth factors and other chemokines [23]. Clearly, protein-GAG interactions play a prominent role in cell–cell interaction and cell growth [24]. 

The time of monolayer 2D cell culture formation takes from a few minutes to a few hours [25]. Three-dimensional cell cultures grow over a few hours to a few days or weeks [25]. Two-dimensional cell culture quality is characterized by high reproducibility of long-term culture [26]. Unfortunately, 3D cell cultures have mostly worse performance and reproducibility, are difficult to interpret and are more difficult to carry out [26]. Moreover, 2D cell cultures do not mimic the natural structure of the tissue or tumor mass while 3D cell cultures mimic in vivo tissue conditions [27]. Two-dimensional cell cultures are deprived of cell–cell and cell–extracellular environment interactions, and have no in vivo-like microenvironment and no “niches” [28]. Three-dimensional cell cultures encourage efficient mass transportation of nutrients and drugs to ensure proper tissue growth and treatment [29,30]. The bioreactor for 3D cell cultures utilizes the central fiber to deliver nutrients and remove waste from an agitated cell suspension. Nutrients were delivered and waste was disposed of in a controlled manner due to the presence of fiber [31,32,33]. However, the development of a cell culture for research began with the discovery of HeLa cells [34,35]. This breakthrough allowed for decades of subsequent research into cell biology due to 2D and 3D cell culturing [36]. The normal spinner flask culture of mammalian cells occurs at densities around 106/mL. Two-dimensional mammalian cell lines or primary cells were suited for the use of 96-, 384-, and even 1536-well plates and are often used for toxicological assays [37]. Nevertheless, 2D systems do not truly recapitulate tissues. Three-dimensional organization allowed multiple measurements from the same tissue region [38,39,40]. Three-dimensional structures can be obtained from dissociated tissue or, importantly, stem cell preparations [41,42,43,44,45,46]. Three-dimensional cell culture models allow the coupling of several different types of tissues and simulate interactions to mimic in vivo conditions [47,48]. In vitro data with cells are compared with in vivo animal data to characterize the in vitro system. In vitro data with human cells in the same system are then used to extrapolate the results to in vivo conditions in humans [49,50,51,52].

To date, the main use of MRI in cellular studies has been in 2D cell cultures or 3D cell cultures to check cell placement. However, 2D cell cultures were difficult objects to measure in an MRI scanner due to the loss of information caused by high amounts of water. Three-dimensional cell cultures are valuable when examining shared cultures or demonstrating a new treatment’s efficacy. Therefore in vitro models of cell cultures are moving from simple 2D to 3D structures to image cellular response [53]. If the score for MCF-7 cells is 0 to 1+, it is called HER2 negative. If the score is 2+, it is called borderline. A score of 3+ is called Her-2 positive. We know that a large number of breast cancers considered HER2-negative have some Her-2 proteins on the surface of their cells. There just are not enough HER2 proteins for the cancer to be considered Her-2-positive. Doctors now consider these cancers Her-2-low. Her-2-low breast cancer has a 1+ score on an IHC test or a 2+ score on an IHC test plus a negative FISH test. IHC test results are most reliable for fresh or frozen tissue samples. IHC tends to be an unreliable test for tissue that is preserved in wax or other chemicals.

Cellular MRI in 3D cell culture is a tool uniquely suited to this task, given its ability to deeply image 3D cell cultures with high temporal resolution and sensitivity. MRI is important to evaluate quantitatively MR parameters such as relaxation times. 

T_1_ is related to the time it takes for the net magnetization to return to its equilibrium position along the Z axis and is called spin–lattice relaxation time [54,55]. T_2_ is the time it takes for the transverse magnetization in the XY plane to return to equilibrium (no spin in the XY plane) and is called the spin–spin relaxation time. T_2_ relaxation emits energy and the signal is proportional to the population difference between the states [54,55].

In the human body, the increased T1 relaxation time corresponds with decreased proliferation and decreased vessel density as seen in suggesting that more extra-cellular extra-vascular space became available [54]. In addition to this, T_1_ in vitro correlates mostly to necrosis of cells and results in shorter T1 thought to be due to the release of complex paramagnetic ions or proteins with the development of cellular necrosis [55].

An important factor in favor of the use of 1.5 T systems is the fact that there is minimal effect on the cultured tumor cells. This state of affairs minimizes the possibility of mistakes in the analyses carried out due to the distant effects of its operation. It is this factor that underlies the use of the medium-field MR system [56,57].

For example, tumor tissues measured in vivo have a longer T_1_ than normal tissue. Generally, older studies have noted both an increase or decrease in T_1_ in vivo relaxation time with treatment [58,59,60,61]. The differences between T_1_ may be due to variability in the tumor model and methods for relaxation time calculation. Moreover, T_1_ in vivo more than T_1_ in vitro can be influenced by a number of parameters such as hardware-related effects, e.g., coil loading and sequence parameters. 

Our result implies that in both types of cells, changes in water relaxation time were attributed to changes in cell hydration. In all six bioreactors with MCF7 cells, we observed significant changes in T_1_ and T_2_ during growing cells [62]. MRI examination of MCF7 cells suggested a possible correlation between the rapidity of the cell culture growth and the progress of the relaxation time changes [63].

The results of these experiments point out wide variations in physical and biological parameters that may be found among a series of individual cancer cell lines of a similar pathological type. The uniform arrangement of the polysulfide fibers ensured that the tissue produced was homogeneous although small intensity variations in the MRI images were observed. These variations suggest that the MRI technique could be used to study the effect of nutrient transport or oxygen gradients on cell profiles. Breast cancer cell development is very intricate and a better understanding of the roles of many more proteins involved in cancer is still needed [64]. 

Thus, for constant membrane permeability, a decrease in the water content should induce a decrease in T_2_. Moreover, the T_2_ of water is sensitive to the structure and the mobility of the surrounding molecules through chemical exchange and dipole–dipole interactions. Therefore, a change in non-aqueous molecular mobility should induce a change in the T_2_ of water. The spin–lattice and spin–spin relaxation times of hydrogen protons of water molecules are a reflection of the interactions of water molecules with macromolecules. In pure liquid water, the T_1_ value for water protons is −3000 ms and indicating rapid motion of water molecules. The majority of cells studied T_1_ suggesting a slower average motion for water molecules [65]. The transverse relaxation time T_2_ is known to be related to the water status in cell compartments which encompasses water content, water mobility and interactions between water and macromolecules [65]. In general, the lower the molecular mobility, the shorter the T_2_ relaxation time, so that the signal from water bound within a polymer matrix decays faster (1–100 ms) than that from free water [65]. 

It has been postulated that slower average motion of water in cells to long- [66] and short-range interactions between water molecules and macromolecular surfaces. MR has been used by a number of investigators to examine the behavior of water molecules in whole organisms [67], normal and cancerous tissues of many kinds [68], in cultured cells [69] during the cell cycle [70], and in solutions of macromolecules [71]. In all cases, the relaxation times were reduced by 3 to 100 times their value in pure liquid water. Analyzing scientific articles on research into cell cultures, it can be concluded that they are the domain of high-field systems [58]. However, it must be said that at present the exact influence of the magnetic field on the human body is not yet known, much less on small and very sensitive structures, such as individual cells.

GAGs are a group of heteropolysaccharides. GAGs consist of polysaccharide chains consisting of repeating disaccharide units. All GAGs, except hyaluronic acid, have a sulfate group and, when attached to core proteins, form proteoglycans (PGs). PGs are characterized by a strongly negative charge of glycan chains and are involved in the selective permeability of cell membranes [72,73,74]. The idea to determine the GAG level by using Gd(DTPA)^2−^ was already proposed in the works [73,74,75,76]. The concept of the method is based on the fact of a negatively charged Gd(DTPA)^2−^ molecule, the spatial distribution of which will be inverse to the GAG concentration. This process was called delayed gadolinium-enhanced cartilage magnetic resonance imaging (dGEMRIC). Gadolinium Gd^3+^ is paramagnetic, which will shorten the T_1_ relaxation time. The process validation itself was described in [77,78]. The authors of work [79] examined the effect of GAG concentration and contrast on T_1_ time for cartilage tissues. They showed that the T_1_ measurement of tissue after administration of a contrast agent can determine the level of GAG, having previously shown that relaxation is linearly dependent on the concentration of Gd(DTPA)^2−^ in the examined tissue. Extensive research on relaxation is included in [80]. The authors tested several available contrast agents from various manufacturers. The study group also included Gd(DTPA)^2−^ used in this study. 

MR researchers [81] assessed several mapping sequences, namely Amide Proton Transfer (APT), DKI and T_2_ for the assessment of the HER-2 gene. Essentially, T_2_ reflects information about water molecules in the tumor microenvironment. This feature is useful in differentiating certain types of cancer. In the cited work, the researchers mentioned the differentiation of tubular adenocarcinoma from non-urethral rectal cancer. The experimental results showed that the longitudinal T_2_ relaxation time was smaller for Her-2 in cells with greater proliferation, while in the case of areas of necrosis, T_2_ was larger. Although the diagnostic efficiency of the APT and T_2_ sequences was higher than that of T_2_ alone, they can be used for non-invasive assessment of EC Her-2 gene expression in the tumor cell microenvironment and at the level of molecular metabolism. The three-dimensional breast cancer cells experimental system provided controlled MRI conditions and allowed for reproducible experimental setup, as well as quantification. Moreover, repeated MRI measurements of the same cells were performed, as the entire bioreactor can be placed inside the magnet and removed without disturbing the cells. Monitoring of Gd-DTPA^2−^ accumulation in 3D cells was observed and the consistent changes in water content resulted in changes in T_1_ and T_2_ values. Moreover, the MRI technique involves cellular administration of the negatively charged contrast agent Gd(DTPA)^2−^, which requires sufficient time to penetrate the high-density cell culture. For the correct evaluation of GAG using MRI of cells, T_1_ measurements need to be conducted at the exact time of cell growth. 

Since GAGs have negatively charged side chains, Gd- DTPA^2−^ is distributed at higher concentrations in areas with lower GAG concentrations. Therefore, a low T_1_ value after administration of the contrast agent indicates a low GAG concentration.

The relaxation time measurement technique used is one of the two most frequently used methods. The saturation recovery (SR) method is a well-documented method in the world literature. Its numerous varieties and modernizations are used for parametric imaging, e.g., of the heart [82,83,84,85] and brain [86,87,88]. The T_1_ time distribution maps shown in the article were based on an application written in the high-level language MATLAB. This package created for scientific and technical calculations is a certain standard in the world of science. Algorithms for selecting a function approximating data have been repeatedly validated in the world of science and technology. The color map imaging itself is also one of the most refined. This package is also one of the most frequently used by the authors of this article [89,90].

In the case of other cell-related techniques, there is, for example, immunohistochemistry, which is considered the gold standard for assessing Her-2 expression. In the field of diagnostic imaging, the imaging of cancer lesions includes computed tomography (CT), ultrasonography (USG), or methods related to nuclear medicine (PET, SPECT) [91]. The gold standard is magnetic resonance imaging, which was also used in this study. The multitude of sequences, their creation in increasing numbers and their modifications mean that this method is gaining new areas of application.

It is not possible in this article to address all imaging methods and data analysis related to cell cultures. Only those that are closest to the content of the article are mentioned.

## 4. Materials and Methods

### 4.1. Cells

The MCF-7 breast cancer cell cultures (American Type Culture Collection, Manassas, VA, USA) were used in the study. EMEM (EBSS) medium (Sigma-Aldrich, St. Louis, MO, USA), 2 mM glutamine (Sigma-Aldrich, St. Louis, MO, USA), 1% NEAA essential amino acids (Sigma-Aldrich, St. Louis, MO, USA) and 10% FBS Fetal Bovine Serum (Biochrom, Berlin, Germany), and penicillin-streptomycin (Sigma-Aldrich, St. Louis, MO, USA) were used to cultivate this cell line. MCF-7 cells were grown under standard conditions: 37 °C, 5% CO_2_, and 95% humidity. Cells were counted using a Muse Cell Analyzer (Merck Millipore, Cambridge, MA, USA).

### 4.2. MRI Equipment

The OPTIMA 360 MRI system from General Electric Healthcare was used for this research. It is an apparatus based on a superconducting magnet with a magnetic field induction of 1.5 T. The parameters of the gradient system, such as the maximum amplitude and speed of gradient increase, were 33 mT/m and 120 T/m/s, respectively. This scanner is equipped with a set of coils for diagnostics of the entire human body. Receiving coils refined to perfection allows for obtaining very good images of the human body. However, they are not sufficient to record the signal from small samples such as cell cultures. Therefore, additional devices were designed to collect signals from small sample volumes. The system used is coupled with medical diagnostic stations equipped with specialized software. This software allows for the analysis of images obtained from the MR system. The set of these devices guarantees high quality of the received images and their proper presentation for analysis by qualified personnel.

### 4.3. Growth of Tumor Cells

We used six bioreactors (*n* = 6) to culture breast cancer cells in 3D geometry. Each bioreactor was inoculated with 0.5 × 10^7^ cells through the rubber septum on the right side port. After inoculation, the bioreactors were perfused using a peristaltic pump (Fisher Scientific, South San Francisco, CA, USA) and maintained in a 5% CO_2_/95% air incubator. Perfusion of the bioreactor was delayed by 4 to 8 h to facilitate adhesion of the cells to the fiber. After that time, the flow was started at a rate of approximately 5 mL/min and was increased over the next days to a rate of 14 mL/min. Cells were grown within the glass tube (inner diameter 10 mm, total length 160 mm) on the multiple porous hollow fibers (inner diameter 700 μm, outer diameter 1300 μm, pore size 0.1 μm) connected at both ends with silicone to the tubing. An immunohistochemistry (IHC) test was performed before MRI measurements to confirm the amount of Her-2 protein in MCF-7 cancer cells. Detection of Her-2 protein was carried out by IHC according to the guidelines published by Wang et al. [92]. For IHC, sections were deparaffinized and antigen retrieval was performed in a pressure cooker at 20 psi for 5 min in citrate buffer (10 mM sodium citrate, 0.05% Tween-20, pH 6.0). Sections were blocked for 30 min with 10% normal goat serum and primary antibodies were applied. Cell nuclei were counterstained with 4′-6-Diamidino-2-phenylindole (DAPI). Fluorescent images were obtained using a Nikon Eclipse fluorescent microscope.

Also, to determine the expression of Her-2 on cells, flow cytometry was performed. The cell suspension was collected and rinsed twice with a blocking buffer. After being suspended in 100 μL blocking buffer, cells (1 × 10^6^) were cultured with FITC-labeled anti-mouse HER-2 antibody for 30 min (4 °C). Then, cells were rinsed several times with fresh PBS, re-suspended in PBS, and assessed by flow cytometry (FCM). The results were analyzed with Flowjo version 10.10 software.

### 4.4. Experimental Conditions of MRI and GAG Measurements

The cell cultures in the hollow fiber bioreactor were placed in the center of the MRI magnet. The prepared samples were scanned using a Fast Spin–Echo (FSE) sequence with an axial projection using a small flexible coil. The following scan parameters were used: field of view (FOV) = 10 × 10 cm; matrix = 320 × 224; NEX = 2.0; slice thickness = 1.0 mm; spacing = 0.5 mm. T_1_ relaxation time measurements were made in steps with the repetition time (TR) as follows: (A) TR = 500 ms, (B) TR = 700 ms, (C) TR = 1000 ms, (D) TR = 1500 ms, (E) TR = 2000 ms, (F) TR = 3000 ms, (G) TR = 5000 ms, (H) TR = 10,000 ms, and (I) TR = 15,000 ms for bioreactor with a fixed echo time (TE) of 3 ms. However, in the case of the T_2_ relaxation time, the TE time fluctuated: TE = 10 ms, (B) TE = 20 ms, (C) TE = 26.2 ms, (D) TE = 42 ms, (E) TE = 68 ms, (F) TE = 85 ms, (G) TE = 102 ms, (H) TE = 130 ms, (I) TE = 160 ms, and (J) TE = 200 ms, TR = 10,000 ms. In addition to this setup, the results of MRI images after adding Gadovist contrast (1 mg/1 mL) were acquired.

The GAG concentration was calculated based on the fixed charge density (FCD) value, which was measured by flushing the culture with Gd(DTPA)2—(Berlex, Montville, NJ, USA). The contrast agent solution was added to the bioreactor at a final concentration of 2 mM Gd ( DTPA)^2−^ to the bioreactor perfusion loop. After 3 h of treatment, the MRI measurement was performed. T1 measurements were performed before and after Gd DTPA^2−^ was added (pre Gd T_1_ and post Gd T_1_) in cells and in media, respectively.

The calculated FCD was converted to GAG concentration according to Lesperance et al. [93]. The concentration of Gd-DTPA^2−^ in cells was derived from T_1_ of cells data according to Equation (1), where T1 media was calculated accordingly to Equation (2). Value R is Gd-DTPA^2−^ relaxivity t 1.5 Tesla and is equal to 3.9 mmol/L/s.

(1) $${\left[Gd{\left(DTPA\right)}^{2-}\right]}_{cells}=\frac{1}{R}\left(\frac{1}{\left(post\;Gd\right)\;T1cells}-\frac{1}{\left(pre\;Gd\right)T1_{cells}}\right)$$

The formula for calculation of [Gd-DTPA^2−^] in cells.

(2) $${\left[Gd{\left(DTPA\right)}^{2-}\right]}_{media}=\frac{1}{R}\left(\frac{1}{\left(post\;Gd\right)T1\;\mathrm{media}}-\frac{1}{\left(pre\;Gd\right)T1\;media}\right)$$

The formula for calculation of [Gd DTPA^2−^] in media.

Tissue FCD was calculated using the [Gd DTPA^2−^] concentration in cells and media Equation (3). The empirical factor of 2 appearing on the right side of Equation (3) is necessary to fit the Donnan-based prediction to biochemically measured FCD [93].

(3) $$FCD_{cells}=-2{\left[Na^{+}\right]}_{media}\left(\frac{{\sqrt{\left[Gd\;{\left(DTPA\right)}^{2-}\right]}}_{cells}}{\sqrt{{\left[Gd{\left(DTPA\right)}^{2-}\right]}_{media}}}-\frac{\sqrt{{\left[Gd{\left(DTPA\right)}^{2-}\right]}_{media}}}{{\sqrt{\left[Gd{\left(DTPA\right)}^{2-}\right]}}_{cells}}\right)$$

${\left[{\mathrm{Na}}^{+}\right]}_{\mathrm{media}}$ is the concentration of Na^+^ ions in the media and is equal to 154 mM. Calculation of tissue GAG content assumes −2 moles of charge per mole of GAG in the cells. The molecular weight of GAG is 502.5 g/mole.

(4) $$\left[GAG\right]=FCD\left(\frac{502.5g/\mathrm{mol}}{2}\right)\times 10^{-3}$$

The formula of GAG calculation in (mg/L). The FCD is the millimolar concentration, and 502.5 is the molecular weight of GAG (g/mol).

MRI data for individual bioreactors are reported as mean ± pixel standard deviation. Owing to the large number of pixels used for these determinations, all quoted differences in MR parameters were found to be statistically significant (*p* < 0.05). Throughout data acquisition, bioreactors were maintained under incubator-like conditions (37 °C, 5% CO_2_/95% air).

### 4.5. MRI and Relaxation Times

The basic assumption of the study was to use an available magnetic resonance system and demonstrate its usefulness for in vitro cell culture studies. The work was carried out on the OPTIMA 360MR device with a field induction of 1.5 T. This system is a clinical examination system equipped with an array of coils for examining the human body. However, the requirements for small sample sizes differ from clinical requirements. It was necessary to design several variants of receiving coils for imaging in a system with a 1.5 T field induction. These circuits were RLC circuits tuned to the resonant frequency of ^1^H hydrogen nuclei for a field of 1.5 T, i.e., 63.885 MHz. These dedicated coils were adjusted to the geometry of the given objects. In the case of spectroscopic tests, the receiving coil was a solenoid coil. This solution provided very good conditions related to the uniformity of the B1 field. Each of these receiver circuits was precisely tuned using RIGOL’s DSA 815 spectrum analyzer. This analyzer, equipped with a tracking generator, measures the characteristics of the analyzed system in the frequency range from 9 kHz to 1.5 GHz. This scope of analysis allows for a thorough examination of the receiving system. The coils were connected to the system using a factory-supplied surface coil adapter.

A single-channel loop coil was used to study cell cultures. The receiving part made of a copper flat bar was connected to the MR system via an adapter. It was provided by the system manufacturer. The proposed structure allowed us to obtain images with relatively high spatial resolution and very good quality.

**Figure 10:**
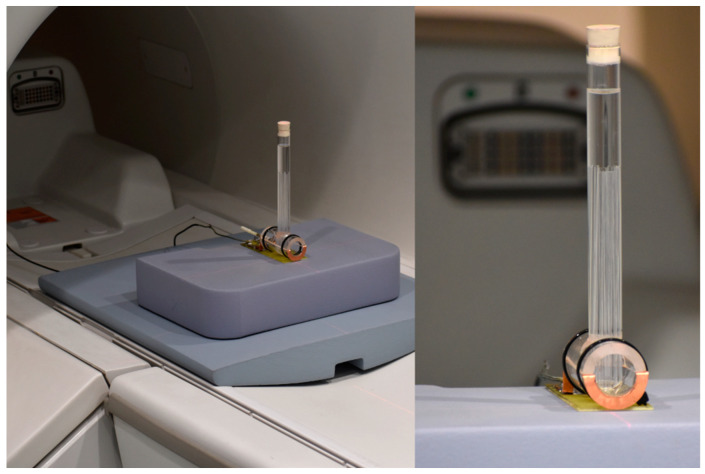
System 1.5 T for clinical trials with a phantom and coil for cell cultures (own study).

**Figure 11:**
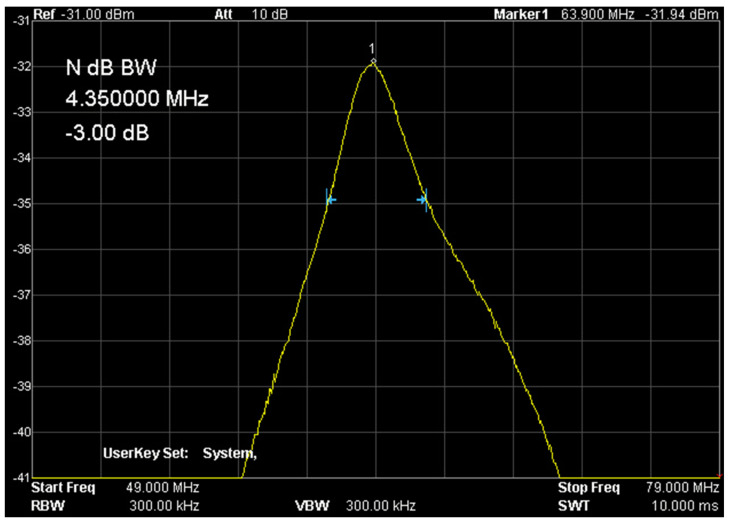
Frequency characteristics of the measuring system (own elaboration). The quality of the presented signal is required and repeated for each sample measurement.

Figure 10 shows the setup of the coil in a 1.5 T magnet, while Figure 11 shows the response of the receiving circuit with frequency characteristics recorded with the RIGOL DSA815 TG spectrum analyzer. The quality of the measuring system was approx. Q = 14.7 and was determined as the ratio of the resonance frequency f0 to the bandwidth with the amplitude decrease by 3 dB–f-3 dB. The resonant load circuit was connected to the system through a dedicated interface ensuring proper wave matching and protection of the entire system and the input stage of the MR system.

For the phantom (Figure 2b), scanning was performed using the following sequence parameters: FOV—5 cm; acquisition matrix—512 × 512; TE = 93.1 ms; TR = 2500 ms; and slice thickness—4 mm. The resolution obtained was 0.1 mm, which is the lower resolution limit of MR microscopy. The lower-left corner of Figure 2 contains data on the scanning parameters and the echo and repetition time values used. This is an image obtained from an MRI system containing system annotations. The measurement time was less than 10 min. The phantom consisted of a test tube with 16 immersed capillaries with an outer diameter of 2.3 mm and a wall thickness of 0.4 mm, filled with demineralized water. The coil used for data acquisition had a rectangular cross-section with the receiving part made of copper tape.

The intensity of the signal in magnetic resonance imaging can be described by the relationship:

$$IS=PD\left(1-e^{-\frac{TR}{T1}}\right)e^{-\frac{TE}{T2}}$$

This relationship connects three material parameters—PD—proton density and T_1_ and T_2_ relaxation times with two parameters that can be set in the MR system—the repetition time TR and the echo time TE. In order to minimize the dependence of the image on the T_2_ time, it is necessary to select the TE time as small as possible. Then, the power expression in the element containing the dependence on time T_2_ will tend to the value 0, and therefore the entire element will tend to the value 1.

$$\underset{TE\to 0}{\lim}PD\left(1-e^{-\frac{TR}{T1}}\right)e^{-\frac{TE}{T2}}=PD\left(1-e^{-\frac{TR}{T1}}\right)$$

Similarly, in the case of eliminating the dependence of the image on the T_1_ time, it is necessary to set the TR value as high as possible. Then, the term containing the dependence on T_1_ will tend to 0.

$$\underset{TR\to \infty }{\lim}PD\left(1-e^{-\frac{TR}{T1}}\right)e^{-\frac{TE}{T2}}=PD\;e^{-\frac{TE}{T2}}$$

In both of the above cases, of course, it is not possible to reach the limit values. In real conditions, the eliminated terms will remain, but their impact on the signal value will be minimized. In the first case, it will be a value less than 1 and will be the factor by which the signal value will be multiplied. In the second case, it will be a value close to 0 and will reduce the signal intensity.

Longitudinal T_1_ relaxation times were determined using the saturation recovery (SR) method. This method involves scanning the object with variable TR time and constant TE time. The TE time should be selected as small as possible, while the range of TR time variability should ensure at least five times the expected T_1_ time. As this time is unknown, the scanning was performed up to the maximum value of the TR time in the system, up to 15,000 ms. Reducing the TR time may increase the error in determining the T_1_ time. Graphs in Figure 8 show the SE method for determining T_1_ and T_2_ relaxation times. The relaxation time T_2_ is determined by changing the echo time TE at a fixed time TR. In this case, it is important to conduct research for as small TE times as possible. This is due to the fact that the maximum value of the signal is important because the T_2_ time is determined from this value.

The second tool necessary to achieve the aim of the study was a program that calculated T_1_ and T_2_ relaxation times. This software was created as an application in the high-level language MATLAB 9.14 (produced by MathWorks, Natick, MA, USA). The basic assumption was to use medical images saved in the DICOM3.0 standard, which are generated by the MRI system. The data contained in these images formed the basis for determining the longitudinal and transverse relaxation times. The software resulted in images of the distribution of T_1_ and T_2_ relaxation times and images of the distribution of the R2 fit coefficient. A detailed description of the operation of the DICOM3.0 file processing algorithm can be presented in several steps.

Step 1: Retrieving directory content data containing images from the DICOMDIR file. This file is a database of images on the media. Its structure, described in the DICOM3.0 standard, contains all the data for the correct identification of sequences and images.

Step 2: Downloading the appropriate images from the media and selecting the analysis area (ROI).

Step 3: Pre-processing of the data to prepare it for approximation. In this step, it is possible to smooth the data by applying a smoothing filter.

Step 4: Data approximation using a mono-exponential or bi-exponential model, and calculation of the T1 or T2 times, depending on the need and the imported data.

Step 5: Presentation of the analysis results in selected graphic windows, including appropriate color profiles. In the case of cross-sectional analysis, only line charts are possible due to the fact that the data from the image profile consists of only one row of pixels. Each T_1_ or T_2_ image is accompanied by an image of the distribution of the R^2^ coefficient, which is a measure of the fit of the approximating function to the set of digital data derived from the images. We managed to obtain a numerical result in which the ROI is the average value, the graph is the image profile along the selected straight line and a map of the time distribution in the selected area along with a histogram of values. The software allows you to export the results to graphic files in popular formats, enabling their subsequent use on a standard PC. The developed application also allows you to download and save both raw data and data analysis along with their parameters to a text file.

### 4.6. A Practical Approach to Signal Analysis and Method Validation

The method of mathematical processing of the signal coming from MRI deserves some explanation. In this case, the signals are images with the resolution possible to obtain during the examination. Color images that are T_1_ or T_2_ maps are a color representation of the distribution of longitudinal and transverse relaxation time coefficients. They are created completely digitally for each pixel separately. Calculating the longitudinal or transverse relaxation time involves finding a function that approximates the measurement data. These data are the brightness values of individual pixels for images obtained for different TR times in the case of longitudinal relaxation and different TE times for transverse relaxation. In the approximation process, it is important to use an appropriate mathematical model consistent with the nature of the phenomenon being described. It often happens that, as a result of various disturbances in the measurement data, a better fit is obtained, determined by, e.g., the R2 coefficient being for a different type of function, which would result from the phenomenon itself.

In this case, the mathematical model was adopted as the one describing the signal intensity value for different times—the equations for T1 and T2 were mentioned above. These are monoexponential models that describe the studied phenomena relatively well. After matching, on the horizontal axis of TR, we can find the time for which the signal intensity value drops to 0.6321 of the maximum value, which approximately corresponds to 63% of this value. For the T2 relaxation time, the time at which the signal intensity drops to 0.3679—approximately 37% of the maximum value—is sought. This is graphically shown in Figure 8. The time values can be read from the chart, but this is subject to a relatively large error. A more accurate method is to calculate the inverse function and calculate the times TR and TE at which the power exponents take the value “−1”, so TR = T_1_ and T_2_ = TE. 

The values read from the DICOM file illustrate different regions of the examined space. They have different T_1_ and T_2_ times, but also different maximum signal intensity values. This may be caused, for example, by different proton densities (PDs) in a given area. The signal intensity is proportional to this PD value. It is therefore necessary to normalize to “1”. 

Below is a function implemented in the MATLAB package to calculate the T1 time based on signal intensity data and TR repetition times. The vector is t1 contains the test data of the curve with time T_1_—2250 ms. The actual R^2^ factors will be lower due to the presence of noise and interference in the signal.


function [f,r2]=timeT1 

%vector of TR times for which image acquisitions were made.

tr=[500, 700, 1000, 1500, 2000, 3000, 5000, 10000, 15000]’; 

% signal intensity vector after normalization different for each image pixel (example data for

% T1 = 2250ms)

is_t1= [ 0.1993 0.2674 0.3588 0.4866 0.5889 0.7364 0.8916 0.9883 0.9987]’; 

% approximation function lan=’a-b*exp(-(c*x)/1000)’; 

% approximation of measurement points using the "fit" function 

%f- contains the values of coefficients a,b,c,

%r2 – contains, among others: value of the curve fitting coefficient to the 

%measurement values R^2 

[f,r2]=fit(tr,is_t1,lan); 

%calculation of T1 time for 63% of values. max. The expression (1-exp(-1))=0.6321 

x=1-exp(-1);

T1=double(-(1000*log((f.a - x)/f.b))/f.c) 

plot(tr,is_t1,’*’); hold on; 

plot(f); 

title(strcat(’T1=’,num2str(double(T1),4),’ms, R^2 =’,num2str(r2.rsquare))); 

xlabel(’TR [ms]’); ylabel(’IS’);grid on, grid minor

end 


For the T_2_ time, the listing will look similar, but the variable describing the approximating function and the function calculating the T_2_ time value will change. The line describing the title of the chart and the description of the horizontal axis will also change.

### 4.7. Subcellular Localization

Cytopainter lysosomal staining (Abcam, Waltham, MA, USA) was examined under the DAPI filter. A blue indicator was warmed to room temperature for 1 h. The working dye solution was prepared by diluting 20 μL of blue indicator with 10 mL of live cell staining buffer. The 108 cells/mL were labeled for 2 h and observed on a fluorescence microscope fitted with a DAPI filter with maximum excitation of 359 nm and maximum emission of 461 nm. Cytopainter lysosomal staining was designed to label lysosomes in live cells in blue fluorescence at Ex/Em = 350/440 nm. Blue staining uses a proprietary lysotrophic dye that selectively accumulates in lysosomes via the lysosome pH gradient. The lysotrophic indicator is a hydrophobic compound that easily permeates intact live cells and is trapped in lysosome cell penetration. Its fluorescence is significantly enhanced upon entering lysosomes. This key feature significantly reduces its staining background.

### 4.8. Multicolor Staining for the Epifluorescence Microscopy

For regular epifluorescence microscopy (non-confocal) to assess cell proliferation, specimens were washed with PBS, then water, and air-dried before imaging. Two fields of view at both low and high magnification were randomly selected and captured for each specimen. DAPI was excited with a 405 nm diode laser light, and a 430 to 470 nm band-pass filter was used to filter emission. 

### 4.9. Statistical Analysis

MRI data are reported as the mean ± SD. Statistical significance was defined as *p* = 0.05. MRI correlations with biochemical data were assessed using Statistica 7.0 (Ucla, Medford, MA, USA). *p* values less than 0.05 were considered significant.

## 5. Conclusions

We have presented a model of 3D in vitro breast cancer cell cultures. MRI results show the possibility of obtaining T_1_ and T_2_ relaxation time over 6 weeks in a 3D culture in a 1.5 T field. We developed an MRI methodology for future study of new breast cancer cell treatments. We hope that our pre-clinical methodology will be helpful for clinic studies and pharmaceutical practices. The research presented here proved that 1.5 T can be adopted for in vitro research. The ability to control the biological, biochemical, and biophysical signals that regulate 3D cell culture confers a number of advantages for this technique. These advantages include (i) the possibility of optimization and standardization, (ii) the production of a larger number of cells, and (iii) improved systems for testing cell responses to a range of experimental parameters. The overall goal was to characterize functional systems with reproducibility. Three-dimensional cell cultivation for MRI detection provides a more physiologically relevant environment for cultures compared to conventional static conditions. The implemented experimental system provided controlled conditions and allowed for reproducible experimental setup, as well as quantification. Moreover, multiple MR measurements of the same cells can be performed, as the entire bioreactor can be placed inside the magnet and removed without disturbing the cells. In this manner, the progress of accumulation of ECM can be observed over the course of time and the consistent changes in water content result in changes in T_1_ and T_2_ values. Moreover, the MRI technique involves cellular administration of the negatively charged contrast agent Gd-DTPA^2−^, which requires sufficient time to penetrate the high-density cell culture. For a correct evaluation of GAG using an MRI of cells, T_1_ measurements need to be conducted at the exact time of cell growth. One important restriction of these techniques is that they measure strong interactions of Gd-DTPA^2−^ with cancer cells and require high cell densities. Since GAGs have negatively charged side chains, Gd-DTPA^2−^ is distributed at higher concentrations in areas with lower GAG concentrations. Therefore, a low T_1_ value after administration of the contrast agent indicates a low GAG concentration.

In conclusion, the possibility of using 1.5 T magnetic field systems for cell culture research and the potential of this scanner to provide information from in vitro studies have been proven. The scanning resolution for FOV = 5 cm and 512 × 512 matrix was achieved at a level of less than 0.1 mm/pixel. Receiving elements were built allowing for the acquisition of data for MRI image reconstruction—this was confirmed by images of a phantom with known structure and geometry. Factory-supplied sequences were modified for the saturation recovery (SR) method, the purpose of which was to determine relaxation times. An application in MATLAB was developed that allows the analysis of these T_1_ and T_2_ relaxation times. This application was used for cell culture research. The relaxation times of cell cultures were determined during 6 weeks of culture. In the first week, the T_1_ time value was 1100 ± 40 ms, while in the sixth week, it was 673 ± 59 ms. For the T_2_ time, the result was 171 ± 10 ms and 128 ± 12 ms

## Figures and Tables

**Table 1:** MCF7 cell culture parameters including age of cells in the week, number of bioreactors and number of cells.

Cells	Week	No. of Bioreactors	Cells ± SD
MCF-7	1	*n* = 6	533 × 10^4^ ± 100
	2	*n* = 6	734 × 10^4^ ± 020
	3	*n* = 6	1451 × 10^4^ ± 060
	4	*n* = 6	4223 × 10^4^ ± 190
	5	*n* = 6	11,622 × 10^4^ ± 140
	6	*n* = 6	63,411 × 10^4^ ± 210

Values are the mean ± SD. The difference in the mean was significant at *p* = 0.05. The mean of cells area increased with development time.

**Table 2:** T1 and T2 relaxation time of MCF-7 cells cultures.

Cell Line	Week	MR Relaxation Time	
		MEN ± SD T_1_ [ms]	MEN ± SD T_2_ [ms]
MCF7	1	1100 ± 40	171 ± 10
	2	1060 ± 56	168 ± 31
	3	911 ± 23	155 ± 33
	4	856 ± 11	149 ± 19
	5	795 ± 31	137 ± 21
	6	673 ± 59	128 ± 12

**Table 3:** GAG concentration.

Week	GAG (mg/mL)
1	1.045
2	1.25
3	1.89
4	1.95
5	2.17
6	2.29

## Data Availability

All data underlying the results are available as part of the article and no additional source data are required.
